# Social and affective support network for public school students experiencing learning problems

**DOI:** 10.1192/j.eurpsy.2023.1556

**Published:** 2023-07-19

**Authors:** P. M. Pacheco, D. R. Molini-Avejonas, M. D. S. Pacheco

**Affiliations:** 1 Speech Language Pathology, University of São Paulo, Vila Velha; 2 Speech Language Pathology, University of São Paulo, São Paulo; 3 Morphology, Universidade Federal do Espírito Santo, Vila Velha, Brazil

## Abstract

**Introduction:**

Adolescence presents itself as a phase of life marked by rapid changes produced by different social contexts and in many cases, it can be configured in a stressful situation. The development of a psychosocial support network is of fundamental importance for adolescents to cope with the pressures of life in challenging situations. Many students, especially living in poor communities, face school problems especially because the curriculum fails to provide relevant knowledge to students in a way it can be meaningful and easier to be taught by teachers and learned by students. When students fail at school usually, they tend to blame themselves and as a result they may develop anxiety, social isolation and even depression.

**Objectives:**

Through the Bioecological Theory of Human Development, we sought to understand the psychosocial support networks of adolescents, whether or not experiencing school problems, considering this to be a challenging event.

**Methods:**

In this research it was used the Five Fields Map, an instrument that evaluated the psychosocial support network for adolescents. The students with and without school problems filled the map in the beginning of the year and then at the end of the same year while facing a school problem as repeating the whole year because of insufficient grades.

**Results:**

The number of relationships between students facing and not facing school problems was not different, however, failing students had fewer relationships in the school-church Mesosystem, fewer relationships in the second and third levels in the first and second moments of data collection, and more relationships in level 5 in the second moment.

**Table:** 

	**School**	**Home**	**Church**	**Public spaces**				
	Rel.	Factor	Rel.	Factor	Rel.	Factor	Rel.	Factor
**Control** **Beginning**	56	5,2	36	4,97	33	6,3	26	6,77
**Control End**	42	5,26	33	4,94	38	6,6	20	6,7
**Total**	98	5,23	69	4,97	71	6,45	46	6,74
**School problem Beginning**	57	5,52	39	5,36	10	6,3	15	5,26
**School Problem End**	45	5,4	31	5,67	10	6,3	10	5,6
**Total**	102	5,47	70	5,51	20	6,3	25	5,53
**TOTAL**	200	5,3	139	5,23	91	6,37	71	6,06

**Conclusions:**

Both group of students showed great strength of proximity in their psychosocial support networks, indicating that it provided sufficient support so that the outcome of the failure experience was positive.

**Disclosure of Interest:**

None Declared

